# Machine learning-based microstructure prediction during laser sintering of alumina

**DOI:** 10.1038/s41598-021-89816-x

**Published:** 2021-05-21

**Authors:** Jianan Tang, Xiao Geng, Dongsheng Li, Yunfeng Shi, Jianhua Tong, Hai Xiao, Fei Peng

**Affiliations:** 1 Department of Electrical and Computer Engineering, Clemson University, Clemson, SC 29634 USA; 2 Department of Materials Science and Engineering, Clemson University, Clemson, SC 29634 USA; 3 Center for Optical Materials Science and Engineering Technologies (COMSET), Clemson University, Anderson, SC 29625 USA; 4 Advanced Manufacturing LLC, East Hartford, CT 06108 USA; 5 Department of Materials Science and Engineering, Rensselaer Polytechnic Institute, Materials Research Center, Troy, NY 12180-3590 USA

**Keywords:** Techniques and instrumentation, Theory and computation, Computational science

## Abstract

Predicting material’s microstructure under new processing conditions is essential in advanced manufacturing and materials science. This is because the material’s microstructure hugely influences the material’s properties. We demonstrate an elegant machine learning algorithm that faithfully predicts the microstructure under new conditions, without the need of knowing the governing laws. We name this algorithm, RCWGAN-GP, which is regression-based conditional generative adversarial networks with Wasserstein loss function and gradient penalty. This algorithm was trained with experimental SEM micrographs from laser-sintered alumina under various laser powers. The RCWGAN-GP realistically regenerates the SEM micrographs under the trained laser powers. Impressively, it also faithfully predicts the alumina’s microstructure under unexplored laser powers. The predicted microstructure features, including the morphology of the sintered particles and the pores, match the experimental SEM micrographs very well. We further quantitatively examined the prediction accuracy of the RCWGAN-GP. We trained the algorithm with computer-created micrograph datasets of secondary-phase growth governed by the well-known Johnson–Mehl–Avrami (JMA) equation. The RCWGAN-GP accurately regenerates the micrographs at the trained time series, in terms of the grains’ shapes, sizes, and spatial distributions. More importantly, the predicted secondary phase fraction accurately follows the JMA curve.

## Introduction

It has been a significant focus in advanced manufacturing and material science to predict the product’s microstructure under a specific processing condition. This is because the material’s microstructure has huge influences on the material’s properties. Conventionally, such knowledge is obtained from trial-and-error experiments. This approach requires extensive and long-term efforts. Substantial progress has been made in the physics-based models that predict microstructures under known governing laws^1–10^. However, these physics-based models usually require high computational costs. It is unrealistic to use physics-based models for real-time microstructure prediction in the advanced manufacturing systems. The data-driven machine learning (ML) approaches^11^ offer potent alternative tools to simulate the material’s microstructure. After training, the data-driven ML algorithms can generate the simulation results almost instantly, without knowing the governing laws.

Deep learning algorithms^12^, especially generative adversarial networks (GANs)^13^, have demonstrated outstanding performances in synthesizing highly realistic images^14–16^. The material’s microstructure is often represented as micrographs from scanning electron microscopy (SEM). Thus, simulating material’s microstructure using GANs has attracted great interests. For example, GANs have been developed to stochastically generate porous media’s solid-void structure^17,18^. A framework was developed to optimize the GAN-generated microstructures with desired material properties^19^. A modified GAN has been used to synthesize porous electrode microstructures for electrochemical energy store devices^20^. In another study, a modified conditional GAN (CGAN) was used to reconstruct metal microstructures based on various cooling methods^21^.

The research mentioned above on GAN-based micrograph synthesis, though successful, is focused on the regeneration of the microstructures from the trained processing conditions. It would be desirable to use machine learning to predict the microstructure under the previously unexplored processing conditions.

In this paper, we develop and demonstrate a novel GAN-based ML algorithm and evaluate the accuracy of such an algorithm for predicting the microstructure under new processing conditions. We demonstrate that our GAN-based algorithm can faithfully predict many aspects of microstructure features under the unknown processing parameters. These microstructural features include the grain sizes, grains’ spatial configurations, and the secondary phase fractions. The prediction of our algorithm is accurate, no matter whether the fundamental governing laws are well-known or even unclear.

## The approach of evaluating RCWGAN-GP for regenerating and predicting material’s microstructure

We name our ML algorithm, ‘RCWGAN-GP’, which means regression-based conditional generative adversarial network with Wasserstein loss function and gradient penalty. The architecture of this algorithm is given in “Methods” section. We built our ML framework based on conditional GANs (CGANs)^22^. Unlike the previous work^21^, we use processing parameters as the numerical conditions, instead of the category condition. This new approach allows the predicted SEM micrographs to be synthesized using the regression of numerical processing parameters. To further improve the prediction accuracy, we implemented and evaluated the Wasserstein loss function with gradient penalty (GP)^23^.

To avoid confusion for the rest of this paper, we call the micrographs that were synthesized using the RCWGAN-GP under the trained conditions, ‘regenerated’ micrographs. We name the micrographs synthesized under new or unexplored processing conditions, ‘predicted’ micrographs.

**Figure 1:**
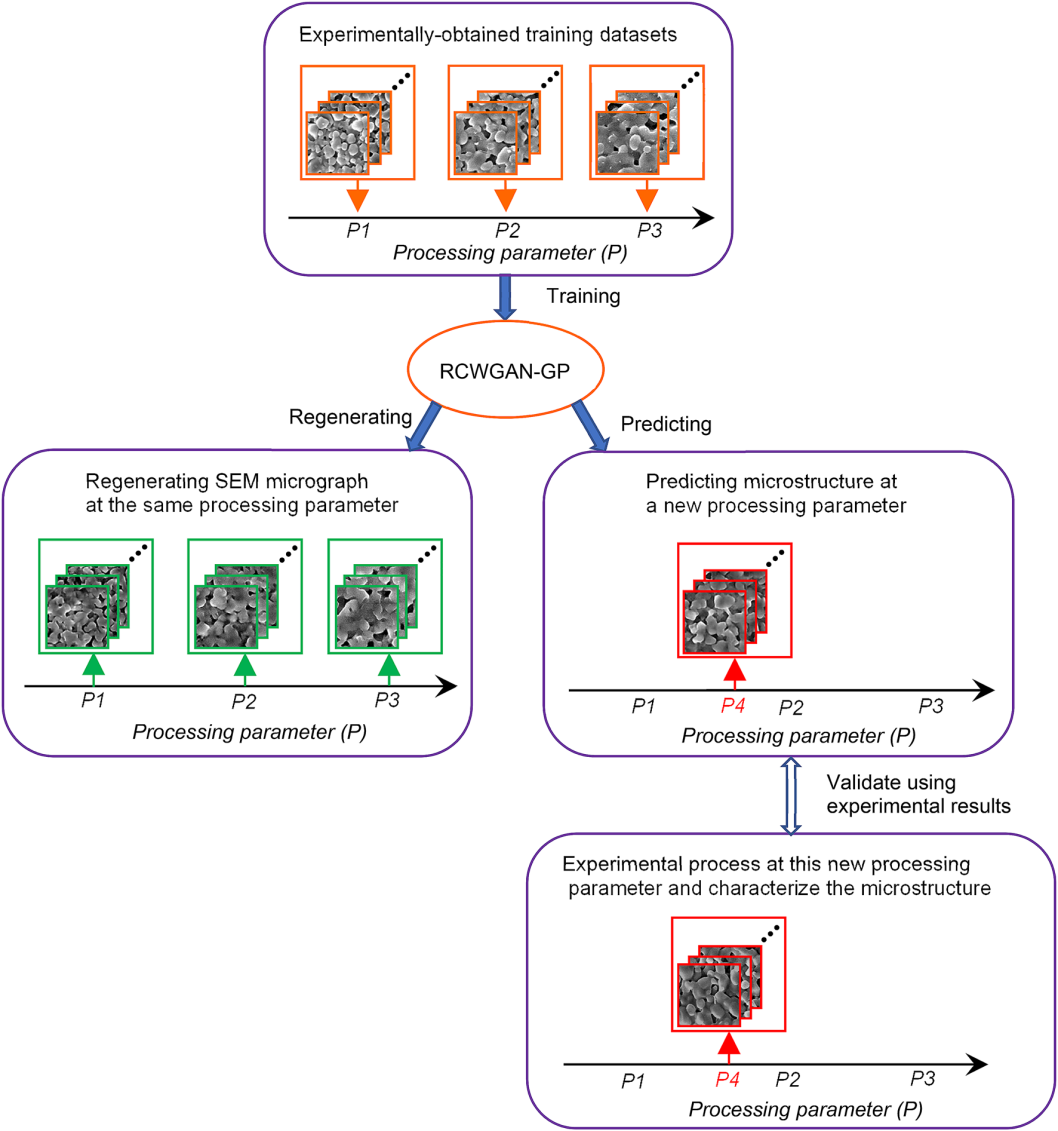
The approach of evaluating RCWGAN-GP for predicting the microstructure of laser-sintered alumina under a new laser powder.

In the first case study, we trained our algorithm with real SEM images that were obtained from laser-sintered alumina samples. The general approach of evaluating the RCWGAN-GP for regenerating and predicting material’s microstructure is given in Fig. 1. In this case study, the laser power is the processing parameter. We collected SEM micrograph datasets under several laser powers. After training, we first let RCWGAN-GP to regenerate the SEM micrographs under the same trained laser power and compare these micrographs to the experimentally obtained ones. Then we used RCWGAN-GP to predict the SEM micrographs at new laser power and compared the predicted SEM micrograph to the experimental results under this new laser power.

In the second case study, we evaluated our algorithm’s accuracy in predicting the statistics of microstructure features using the computer-created micrograph dataset. The computer-created micrographs have simple microstructure features. Thus, the statistics of the microstructural features of the training micrographs and RCWGAN-GP-synthesized ones can be easily analyzed using a computer program. The training micrograph dataset represents the secondary phase growth over a series of time, following the classic Johnson–Mehl–Avrami (JMA) Eq. ^24^. For this case, the processing parameter of interest is normalized time (*t*/*τ*). Thus, time was used as the numerical condition for training.

## Results and discussion

### Compare the performances of CGAN, CWGAN, and CWAN-GP in regenerating SEM micrographs

We trained the ML algorithms using real SEM micrographs obtained from laser-sintered alumina. These SEM micrographs were divided into 5 subsets corresponding to 5 different laser powers (*P* = 1.4 W, 1.5 W, 1.7 W, 1.8 W, and 1.9 W). The processing parameter of interest is the laser power. The experimental procedure is described in our previous study in details^25^. The training dataset consists of SEM images taken from the samples sintered under different laser powers, while the other processing parameters (*e.g.* laser scanning speed, defocus distance, and material parameters) are kept constant. The SEM micrographs under laser power 1.6 W were not used for the ML training but saved for evaluating the prediction accuracy. There were 2080 images in each subset. Each image is 128 × 128 pixels in size, and the real sample area was 6.4 × 6.4 μm^2^.

**Figure 2:**
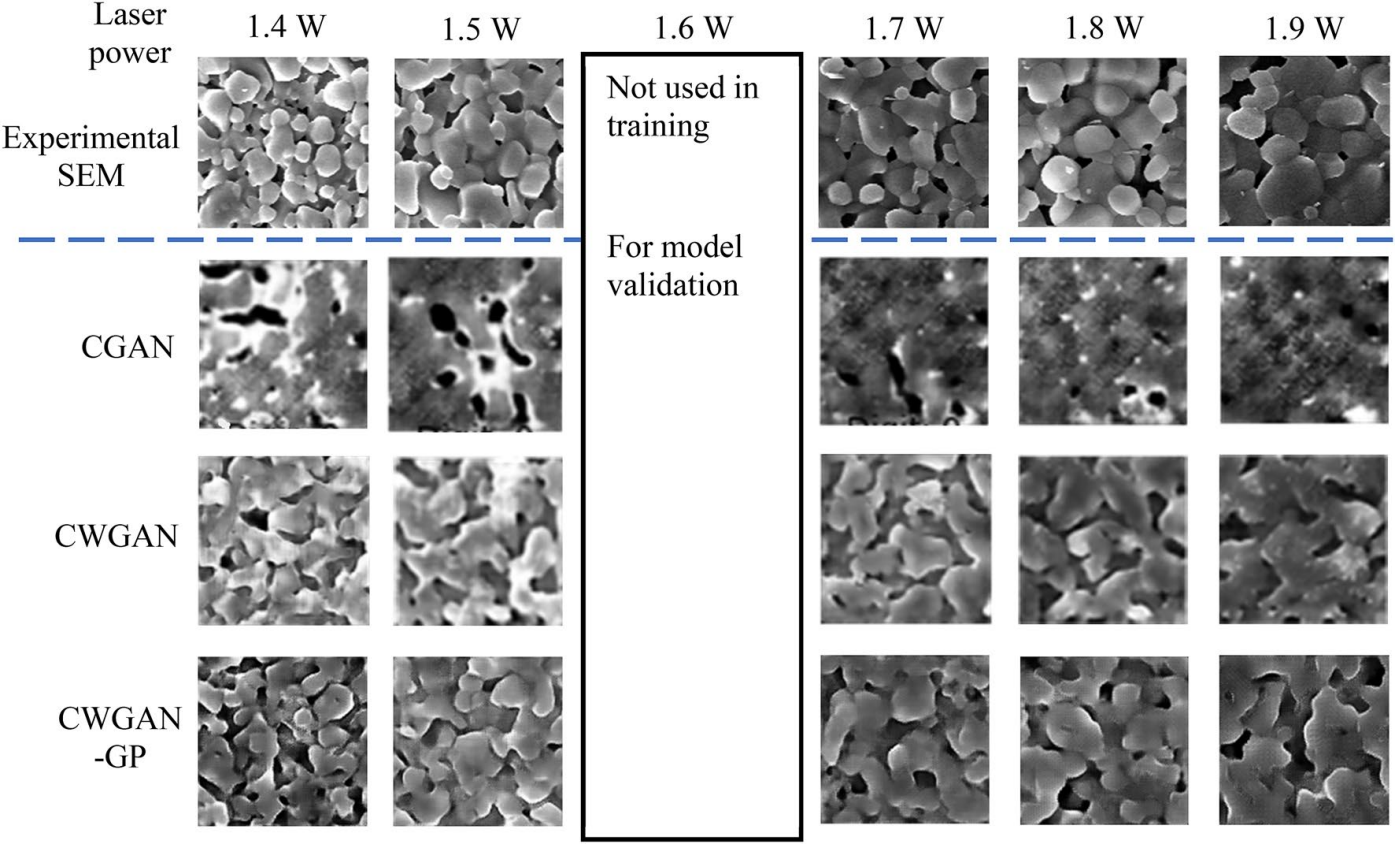
The examples from the real SEM image dataset, and the regenerated micrographs using CGAN, CWGAN, and CWGAN-GP.

We initially build our ML network based on CGAN, since CGAN allows the combining of SEM data with processing parameter. However, after the initial study, we found that no matter how we optimized the algorithm, the results were not satisfactory. In a typical SEM micrograph of laser-sintered alumina, we evaluated the critical microstructure features, which are the the morphology,. During laser sintering, when the laser power increased, the obtained alumina had larger size, lower porosity, and higher relative density. As shown in Fig. 2, if we use CGAN for the SEM micrograph synthesis, we can only observe the decrease of porosity with increasing laser power. The microstructural features of particles were lost.

Then we improved the CGAN with the Wasserstein loss function^26^. The improved algorithm is known as conditional Wasserstein GAN (CWGAN). Great improvement was found for CWGAN as shown in Fig. 2. The microstructural features of grains and pores can be observed in the synthesized SEM micrographs. However, the grain boundaries and 3D configuration of the grains were still unclear. This makes the regenerated microstructures have larger average particle size than the real SEM. These defects might originate from the weight clipping technique used in the CWGAN^26^. Finally, we replaced weight clipping with gradient penalty^23^, and developed CWGAN-GP algorithm for our simulation and prediction.

This CWGAN-GP showed best accuracy in synthesizing SEM micrographs. The regenerated SEM micrographs have clear grains and pores. The grain size, porosity and relative density match the real SEM micrograph very well. The CWGAN-GP also captured the trend that the particles were growing larger and being sintered together when the laser power increased.

### Develop RCWGAN-GP to predict SEM images of laser-sintered alumina under a new laser power

Since CWGAN-GP showed the best performance in synthesizing SEM micrographs as shown in Fig. 2, our further research was based on CWGAN-GP. We developed RCWGAN-GP by making the network implicitly regress the conditional probability distribution of the training data against the input condition. This RCWGAN-GP was inspired by the research on the regression-based CGANs^27,28^. A study found that the regression of CGANs exhibited competitive or even better performance in comparison with other regression algorithms^29^.

**Figure 3:**
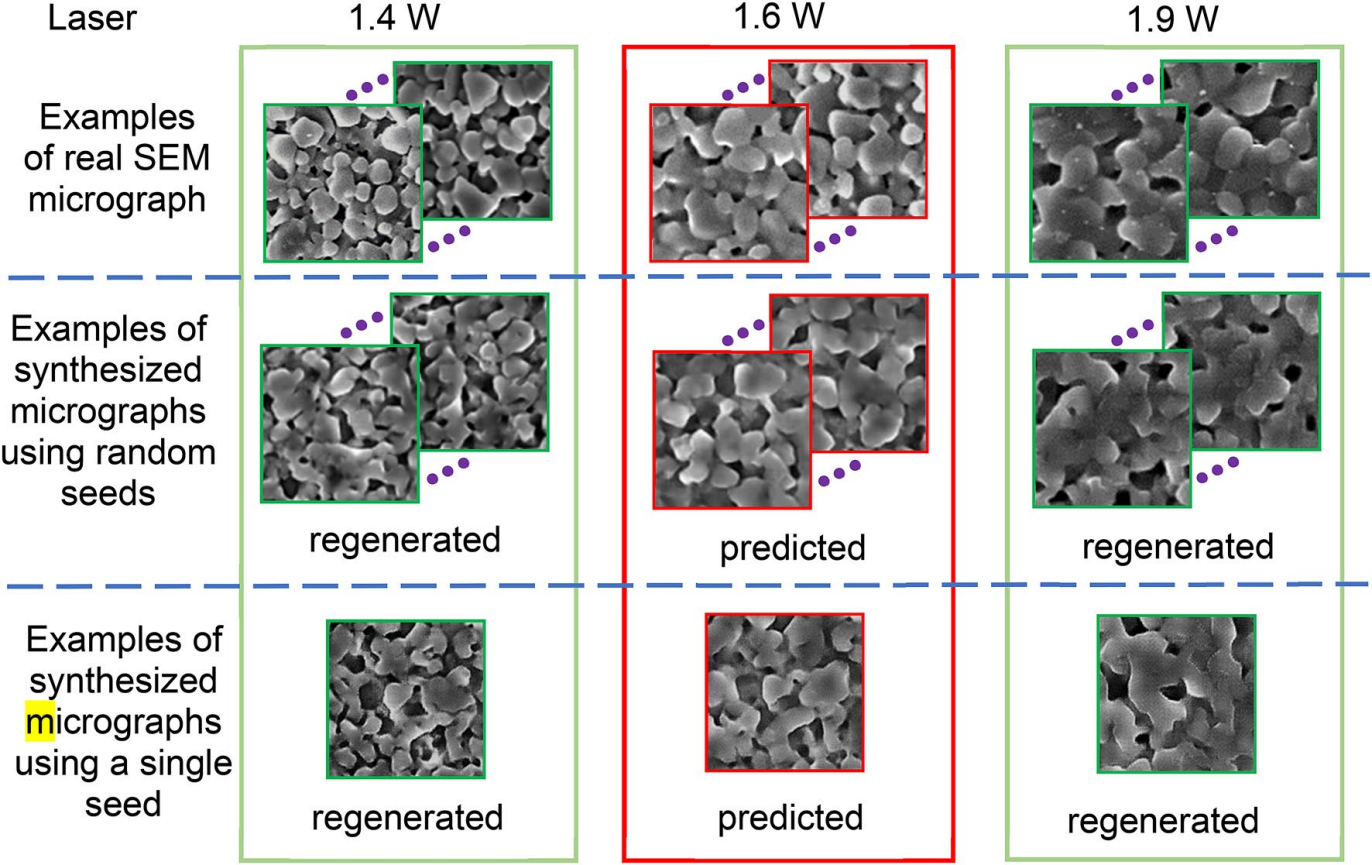
The examples of regenerated and predicted SEM micrographs using the RCWGAN-GP. For the laser power 1.6 W highly imitate the real SEM micrographs, in terms of microstructural features such as particle and pore morphologies .

We are interested in evaluating the performance of the RCWGAN-GP in predicting the microstructure of materials when the underlying mechanisms of the process-microstructure relation are unclear. First, we regenerate the SEM micrographs under the trained laser powers. As shown in Fig. 3, at the trained laser powers, *e.g.*, 1.4 W and 1.9 W, the ML model regenerates highly realistic SEM micrographs, in terms of the morphology of the particles and pores.

Then we use the RCWGAN-GP to predict the microstructure of laser-sintered alumina at the laser power of 1.6 W. Predicting the microstructure of laser-sintered alumina is challenging, since the processing mechanism is still unclear. In our previous study, we demonstrated ultra-fast, solid-state laser sintering of alumina^25^. In that study, the resulting alumina’s microstructure deviates from the typical sintering master curve^30,31^. Due to the complexity of the ultra-fast laser sintering mechanisms, it is difficult to establish a physics-based model that can predict the laser sintered alumina’s microstructure features.

The training dataset did not include the micrographs under this laser power of 1.6 W. In the meantime, we collected the real SEM micrographs of alumina sintered under 1.6 W laser power to evaluate the prediction accuracy. This comparison is given in Fig. 3. The predicted SEM micrographs at 1.6 W laser power faithfully imitate the real SEM micrographs, in many aspects of microstructural features, such as the morphology of particles and pores These key microstructural features are very important to predict the material properties in the future studies. In addition, the predicted microstructure features also accurately reflected the trend of the influence of laser power. In our previous study, we showed that a higher laser power resulted in a larger particle size, larger relative density, and lower porosity^25^. The predicted SEM micrographs under 1.6 W of laser had particle size and relative density that are clearly larger than those of 1.4 W, but smaller than those of 1.9 W.

We examined both situations of synthesizing SEM micrographs using random seeds and a single seed. When random seeds were used, the synthesized SEM micrographs show similar microstructure features for one laser condition. We explored the latent space of the generated images to rule out the possibility of image-generation as a result of “memory-effect”. As shown in Fig. 3, when we use a single seed to synthesize SEM micrographs, the regenerated and predicted images morph continuously from one microstructure to the other, indicating that the laser power-microstructure relationship are indeed “learned” by the neural network.

**Figure 4:**
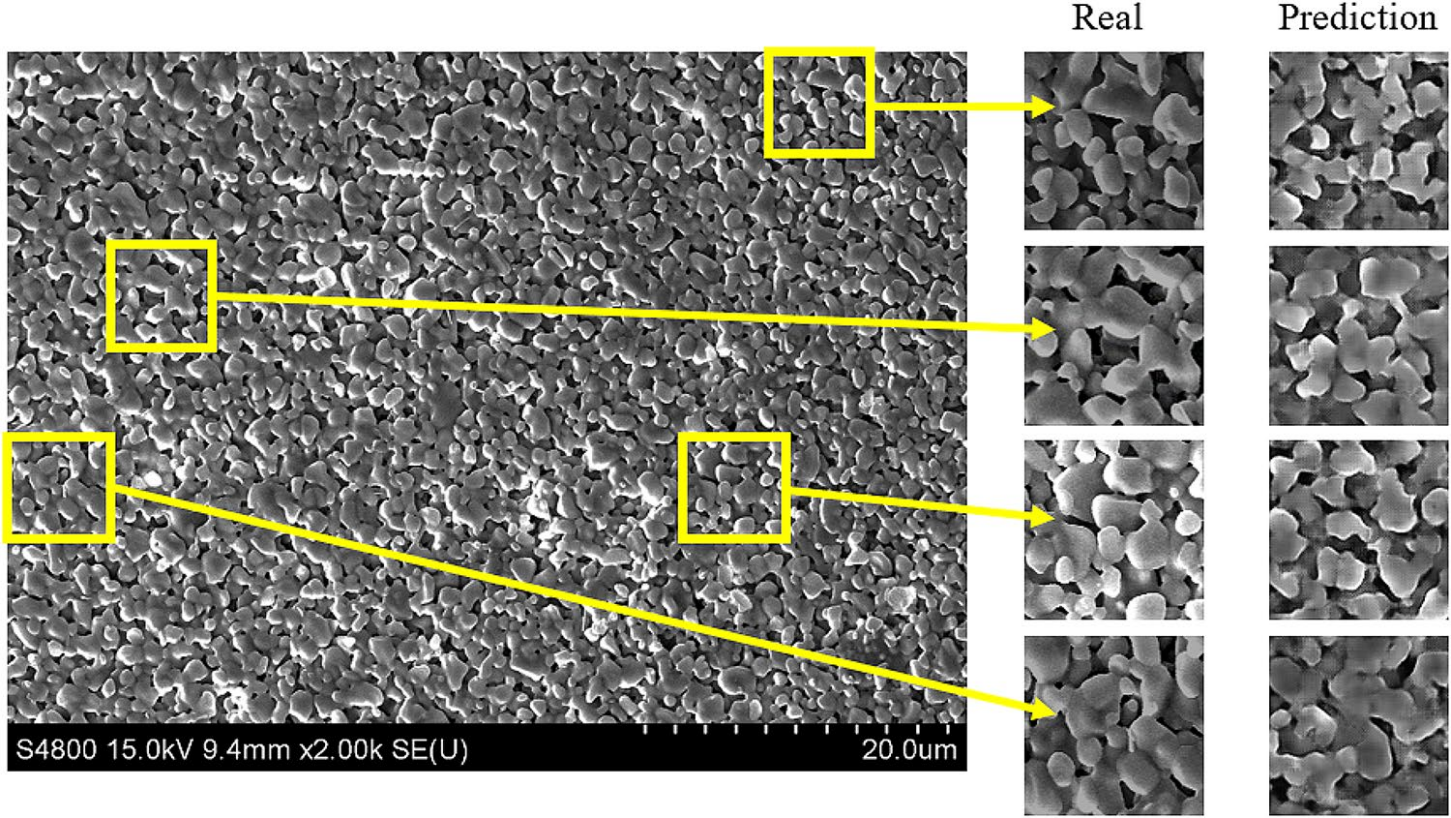
The large domain SEM image of an alumina sample sintered under 1.6 W, with small domain of SEM magnified from the large one and the corresponding predicted SEM images using the RCWGAN-GP.

It is worth noting that even within one experimentally obtained SEM micrograph, there are variations in the microstructure at different locations. We found that the RCWGAN-GP can also predict such microstructural variation, as shown in Fig. 4. On the left side of Fig. 4 is a full-size real SEM micrograph of laser-sintered alumina at 1.6 W. Several segmented images of 128 × 128 pixels are extracted from the full-size SEM micrographs as representatives. On the right to the column of real image segments, are the predicted images using RCWGAN-GP from random seeds. This variation is an important characteristic of laser sintered ceramics resulted from the nature of the laser sintering process. In our training datasets of the laser power of 1.4 W, 1.5 W, 1.7 W, 1.8 W, and 1.9 W, we observed microstructure variation. This is the reason that the predicted results also present this microstructural variation, which was learned from the training data. Apart from the visual similarity, it is another evidence that the RCWGAN-GP is capable of predicting microstructures under unexplored values of a processing parameter.

Thus, we demonstrate that RCWGAN-GP can regenerate realistic SEM micrographs under the trained laser powers and faithfully predict the SEM micrographs of alumina under an unexplored laser power. The predicted micrographs match the experimentally obtained micrographs in particle size, particles’ spatial configuration, and porosity. These are the key microstructure features that determine the mechanical properties of the alumina ceramic.

### Prediction of the simulated microstructure during secondary phase growth

In the above case study, it is difficult for us to have great statistical comparisons of particle and pore sizes between RCWGAN-GP predicted SEM micrographs and experimentally obtained ones. This is because we usually determine the statistics of grain and pore sizes using manual methods on the SEM micrographs^25^. We were not able to quantitatively characterize the grain size and pore size distributions because the boundaries are not clear. To our best knowledge, there is no reliable benchmark algorithm that can automatically and accurately extract the above-mentioned statistical information from the porous alumina SEM micrographs.

**Figure 5:**
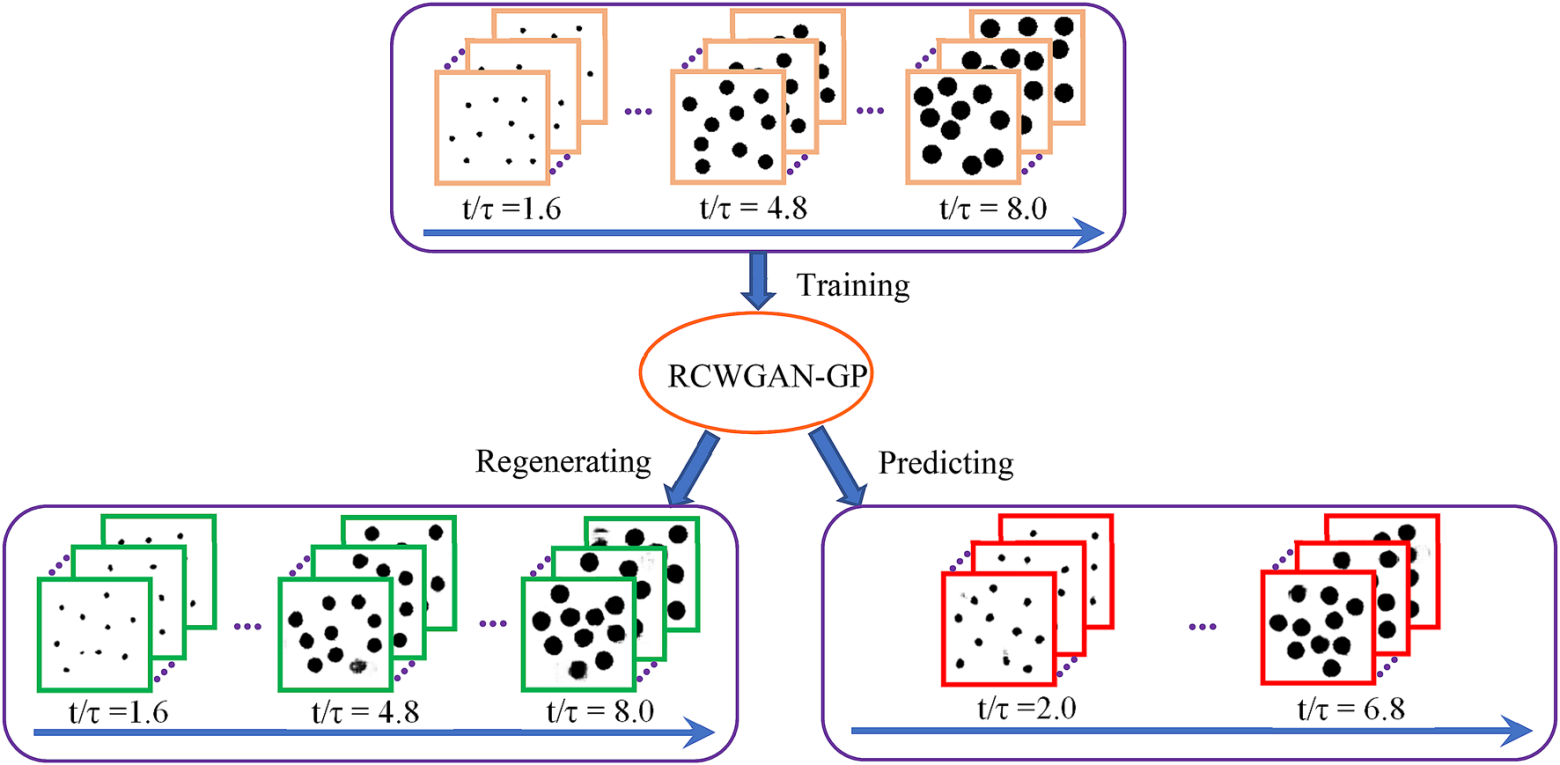
Using computer-generated micrographs to evaluate the accuracy of the RCWGAN-GP in regenerating and predicting microstructure of secondary phase grain growth. The processing parameter are a time series, t/τ = 1.6, 2.4, 3.2, 4.0, 4.8, 5.6, 6.4, 7.2 and 8.0. After training, RCWGAN-GP can regenerate micrographs that have similar microstructure features at the trained time series. We use RCWGAN-GP to predict the microstructure at a new set of time, (t/τ) = 2.0, 2.8, 3.6, 4.4, 5.2, 6.0, 6.8, and 7.6. The predicted micrographs showed the expected microstructure features during the secondary phase grain growth.

To obtain the statistical comparison between the real and RCWGAN-GP-predicted micrographs, we used a computer program to create an idealized and simplified training dataset of micrographs. These micrographs can be easily recognized and analyzed to obtain the statistics of microstructural features. The training dataset contained 9 subsets of micrographs, with each subset having one thousand micrographs. For each subset of micrographs, a value of normalized time (*t/τ*) was assigned as the processing condition. In the same subset, all micrographs contained 10 randomly distributed, non-overlapping, same-sized black circles, as shown in Fig. 5. These black circles represented the secondary phase grains. To represent grain growth , the sizes of black circles were larger at a larger value of time. We set the areal fraction (*f*) of the secondary phase at time (*t/τ*) to follow JMA equation:

1 $$f = C_{1} *\left( {1 - exp \left( { - C_{2} \left( {\frac{t}{\tau }} \right)^{2} } \right) } \right)$$

where *τ* represents a characteristic time constant. *C*_*1*_ and *C*_*2*_ are two material-related constants. In this study, we arbitrarily set *C*_*1*_ as 0.28, and *C*_*2*_ as 0.03.

**Figure 6:**
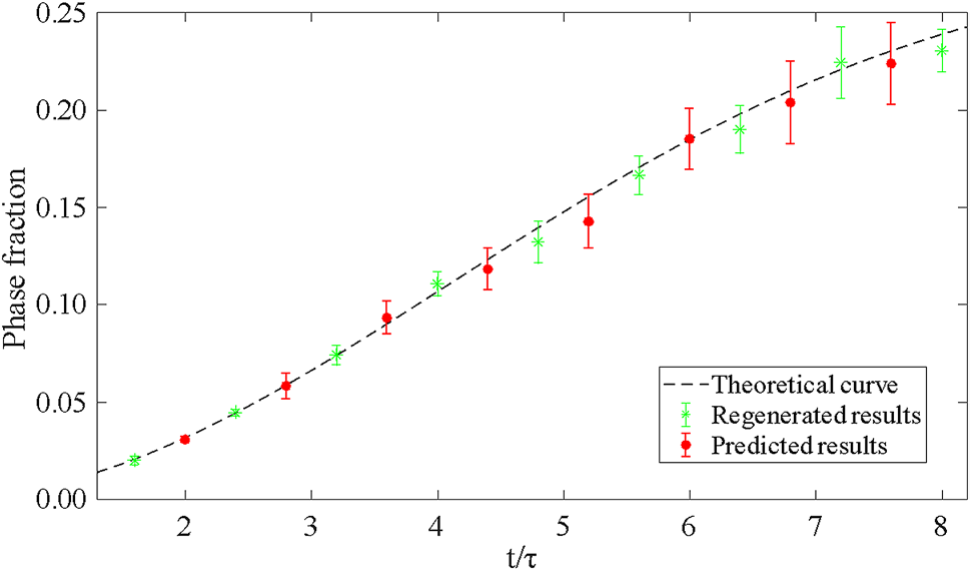
The fraction of the secondary phase of the regenerated and predicted SEM micrographs using RCWGAN-GP. The RCWGAN-GP-predicted the secondary phase fraction fits the JMA equation very well.

After training, we use the RCWGAN-GP to synthesize the micrographs at the exact same time values (*t/τ*) as the training dataset. From Fig. 5, we can see that the regenerated micrographs have the same features as the training dataset, in the number, size, shape and spatial distributions of the secondary phase grains. Each micrograph contains 10 isolated circular dots. The sizes of the dots are approximately equal in each regenerated micrograph, and similar to the training micrograph. If we plot the areal fraction of the secondary phase vs. time, as shown in Fig. 6, we found that regenerated micrographs follow Eq. (1) quite well. We also observe some defects in the RCWGAN-GP regeneration. Sometimes there are vague shadows in the regenerated micrographs. The dots are not perfect circular either. These defects may be caused by the unoptimized hyperparameters or insufficient dataset size.

Then we use RCWGAN-GP to predict the microstructures at a new set of values of time (*t*/*τ*). Ten micrographs were predicted for each (*t*/*τ*) values. The examples of the predicted micrographs are also shown in Fig. 5. Very interestingly, after regression, we found that the ML algorithm can quite accurately predict the microstructure for unexplored time conditions, in terms of the number, size, shape and spatial distributions of the secondary phase grains. For example, as shown in Fig. 5, predicted micrographs at *t/τ* = 2.0 contain ten equal-sized, random-positioned dots that are larger than *t/τ* = 1.6, but smaller than *t/τ* = 4.8.

As shown in Fig. 6, if we plot the total secondary phase fraction vs. time, we found that the predicted phase fraction follows Eq. (1) very well. Meanwhile, we observed similar defects in the predicted micrographs to those in the regenerated micrographs. We observed the increase of the standard deviation at the later time (*t/τ*), for both regenerated and predicted micrographs. It might originate from the irregularity of the shape of the regenerated or predicted dots. Some dots can have protuberances, while some miss pixels. The irregularity is proportional to the size of the dots. Thus, the standard deviation increases when the size of the dots increases.

In conclusion, for the computer-created training dataset, the RCWGAN-GP can realistically regenerate the micrographs at the trained time points. This algorithm can also faithfully predict the microstructures at a new time series, with good accuracy in many microstructure features, such as the number, size, shape, and spatial distributions of the secondary phase grains.

## Conclusion

In this work, we develop a machine learning algorithm RCWGAN-GP, that not only can regenerate highly accurate micrographs for known processing parameters, but also can predict microstructures for unexplored processing parameters. This machine learning algorithm is regression-based conditional generative adversarial networks with Wasserstein loss function and gradient penalty.

We trained the algorithm with a real SEM dataset obtained from laser-sintered alumina. Under different laser powers. After training, the RCWGAN-GP regenerates highly realistic SEM micrographs under the trained laser powers. The particle size, porosity, and spatial distributions of the particles were similar to the features in real SEM micrographs. With the increase of laser power, changes in microstructures, such as particle growth and porosity decrease, are observed in regenerated SEM micrographs. Remarkably, the predicted micrographs under a new laser power are visually similar to the experimental results, in many aspects of microstructural features. The predicted microstructure accurately represented the trend of the effect of laser power on the microstructure evolution. The prediction results also capture the microstructure variation at different locations which was a feature in the training dataset.

Then, we quantitatively demonstrate the regeneration and prediction capability of the algorithm with computer-created micrograph dataset. The computer-created micrographs are simplified for the statistical analysis of the microstructural features. The computer-created dataset has artificial micrographs that represent the secondary phase growth process at a time series. After training, the algorithm regenerates and predicts micrographs with high accuracy in terms of microstructural features. The predicted secondary phase fraction fit the JMA curve very well. This indicates that the algorithm can accurately capture the relation between the time and the secondary phase fraction.

## Method

### Architecture of RCWGAN-GP and the training process

**Figure 7:**
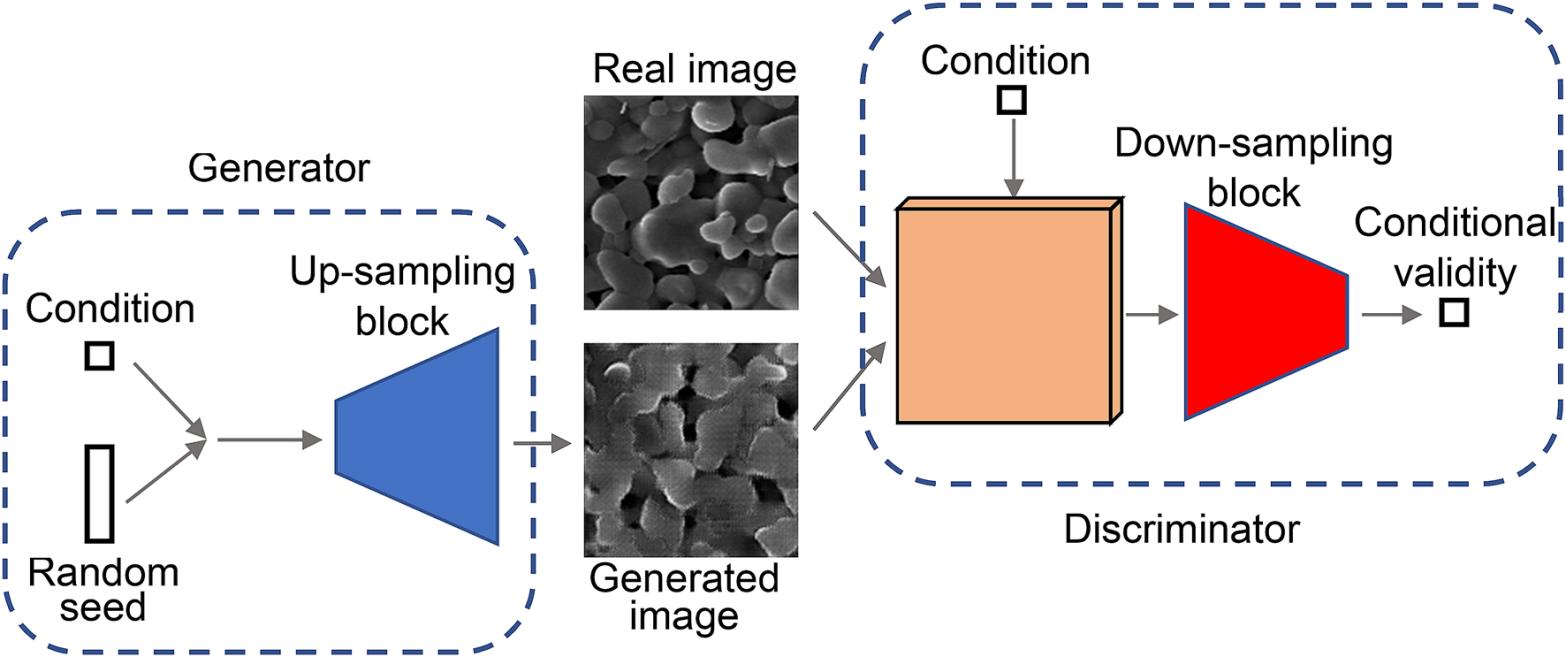
The structure of the RCWGAN-GP.

The architecture of the generator and the discriminator of RCWGAN-GP of this paper is shown in Fig. 7. The generator takes two inputs. One is a random vector whose dimension is one hundred. It is used as a seed for image generation. The other one is the condition, which is a single scalar. The seed is expanded by a dense layer and then reshaped to 8 × 8 × 128. The condition is also expanded by a dense layer and then reshaped to 8 × 8 × 16. The following up-sampling block consists of four transposed convolutional layers and two convolutional layers. In the transposed convolutional layers, the kernel sizes of these layers are 5 × 5, and the strides are 2. The numbers of channels are 256, 128, 64, and 32. The activation functions are leaky rectified units (LeakyReLUs). LeakyReLUs are defined as following:

2 $$f\left( x \right) = max\left( {x, \alpha x} \right)$$

where α is set to be 0.2. Both convolutional layers use 3 × 3 kernels and 1 stride. The numbers of channels are 16 and 1, respectively. They employ hyperbolic tangent functions as their activation function. The output is a 128 × 128 × 1 image. The discriminator has a similar but inverted structure. The condition is first expanded, reshaped to 128 × 128 × 1, and concatenated to the input image, either real or generated. Then five convolutional layers perform down-sampling. The kernel sizes are 5 × 5, and strides are 2. The numbers of channels are 16, 32, 64, 128, and 256. The activation functions are LeakyReLUs. The output layer has a single node to output a scalar.

When training the generator, the loss function is defined as:

3 $$L_{g} = - E_{{\tilde{x}|y \sim P_{g} }} \left[ {D\left( {\tilde{x},y} \right)} \right]$$

where $\tilde{x}$ is a generated image, $y$ is the condition, and $P_{g}$ represents the generator distribution. $D\left( {\tilde{x},y} \right)$ is the output of the discriminator.

When training the discriminator, the loss function is similar to WGAN-GP^26^:

4 $$L_{D} = E_{{\tilde{x}|y \sim P_{g} }} \left[ {D\left( {\tilde{x},y} \right)} \right] - E_{{x|y \sim P_{r} }} \left[ {D\left( {x,y} \right)} \right] + \lambda E_{{\tilde{x}|y \sim P_{{\hat{x}}} }} \left[ {\left( {\left\| {\nabla_{{\hat{x}}} D\left( {\hat{x},y} \right)} \right\|_{2} - 1} \right)^{2} } \right]$$

where $x$ is a real image, and $P_{r}$ represents the real data distribution. The third term is the gradient penalty term. $P_{{\hat{x}}}$ is uniformly sampling along the straight line between $\tilde{x}$ and $x$. Thus, $\hat{x}$ is calculated as following:

5 $$\hat{x} = \epsilon x + \left( {1 - \epsilon } \right)\tilde{x}$$

where $\epsilon$ is a random number that uniformly distributes on [0,1]. $\lambda$ is a hyperparameter that controls the weight of the gradient penalty loss. In all experiments, it is set to be 10.

Training details are described below. The implementation and training of the algorithm were based on Keras^32^. We used the Adam optimizer^33^ when training the generator as well as the discriminator. The learning rate is 2E−4 for the generator and 1E−4 for the discriminator. $\beta_{1}$ is 0.0 and $\beta_{2}$ is 0.9 for both the generator and the discriminator. We used mini-batch gradient descent method, where the size of one mini-batch is 128. We train the algorithm for 700 epochs on Palmetto, the cluster of Clemson University. It takes 5 h to complete on 8 CPUs and 1 GPU (Nvidia P100).

### Use computer to generate idealized SEM micrographs that follows JMA equation

Each computer-generated SEM micrograph had ten black dots of the same size randomly distributed on a white background. The black dots represented the growing second phase solids over time, while the white background represents the primary phase. The area of the white background is 128 × 128 pixels. The area of one black dot is 128 × 128 × *f*/10 pixels, where *f* is the fraction of the secondary phase. To simplify the problem, the black dots were not overlapping with each other. The distance is constrained to be at least 1.5 times the diameter of the dots. The positions of the ten black dots are randomly picked by the Monte Carlo method. One thousand images with the same phase fraction are simulated to form a subset, which is labeled by the value of (*t*/*τ*). Nine subsets form the training set.

Since the outputs of our network were gray scale images rather than binary images, we binarized all the generated images before calculating the phase fractions. The phase fraction (*f*) of each micrograph is calculated by dividing the number of black pixels by the total area. Then we average the phase fractions over the samples that have the same labels of time (*t/τ*).

### SEM image acquisition

During the sintering experiment only laser power is changed while all the other processing conditions were kept as constants. Sintered alumina was characterized using SEM to study the influence of laser power on microstructure. The detailed processing procedure is reported in our previous work^25^. During a typical laser sintering experiments, alumina paste is casted on a fused silica substrate, using doctor’s blade, with a thickness of about 500 μm. The green body was sintered using a CO_2_ laser (firestarv20, wavelength 10.6 μm, SYNRAD, Inc., WA). Five different laser powers were selected (*i.e.* 1.4 W, 1.5 W, 1.6 W, 1.7 W, 1.8 W and 1.9 W), while the other processing parameters were kept the same.

We took eight SEM images at different locations along the center of laser-scanned track. The magnification is kept as 2000X. Each image had 896 × 1280 pixels. To increase the SEM dataset size, we segmented the SEM images into smaller images of 128 × 128 pixels. This segmentation size was carefully chosen. If it was too large, the number of samples would be insufficient for training. If it is too small, there would be an insufficient number of particles in each small image, making the dataset unrepresentative of the microstructure features, such as particle size, porosity, and relative density. After segmentation, there were 560 images for each laser power. To further augment the dataset, we rotate every image by 90, 180 and 270 degree so that the number of images were quadrupled. This image augmentation does not only increase the number of samples, but also prevent the algorithm from overfitting^34^.
